# Landscape ethnoecological knowledge base and management of ecosystem services in a Székely-Hungarian pre-capitalistic village system (Transylvania, Romania)

**DOI:** 10.1186/1746-4269-11-3

**Published:** 2015-01-07

**Authors:** Zsolt Molnár, Krisztina Gellény, Katalin Margóczi, Marianna Biró

**Affiliations:** Institute of Ecology and Botany, MTA Centre for Ecological Research, Hungarian Academy of Sciences, Alkotmány u. 2-4, H-2163 Vácrátót, Hungary; University of Szeged Department of Ecology, Közép fasor 52, H-6726 Szeged, Hungary

**Keywords:** Central Europe, DPSIR framework, Ecosystem functions, Ecosystem regeneration, Habitats, Resource management, Traditional ecological knowledge, Village laws, 16-19^th^ centuries, Sustainability

## Abstract

**Background:**

Previous studies showed an in-depth ecological understanding by traditional people of managing natural resources. We studied the landscape ethnoecological knowledge (LEEK) of Székelys on the basis of 16-19^th^ century village laws. We analyzed the habitat types, ecosystem services and sustainable management types on which village laws had focused.

**Methods:**

Székelys had self-governed communities formed mostly of “noble peasants”. Land-use was dominated by commons and regulated by village laws framed by the whole community. Seventy-two archival laws from 52 villages, resulting in 898 regulations, were analyzed using the DPSIR framework. Explicit and implicit information about the contemporary ecological knowledge of Székelys was extracted. We distinguished between responses that limited use and supported regeneration and those that protected produced/available ecosystem services and ensured their fair distribution.

**Results:**

Most regulations referred to forests (674), arable lands (562), meadows (448) and pastures (134). Székelys regulated the proportion of arable land, pasture and forest areas consciously in order to maximize long-term exploitation of ecosystem services. The inner territory was protected against overuse by relocating certain uses to the outer territory. Competition for ecosystem services was demonstrated by conflicts of pressure-related (mostly personal) and response-related (mostly communal) driving forces. Felling of trees (oaks), grazing of forests, meadows and fallows, masting, use of wild apple/pear trees and fishing were strictly regulated. Cutting of leaf-fodder, grazing of green crops, burning of forest litter and the polluting of streams were prohibited. Marketing by villagers and inviting outsiders to use the ecosystem services were strictly regulated, and mostly prohibited. Székelys recognized at least 71 folk habitat types, understood ecological regeneration and degradation processes, the history of their landscape and the management possibilities of ecosystem services. Some aspects of LEEK were so well known within Székely communities that they were not made explicit in village laws, others remained implicit because they were not related to regulations.

**Conclusions:**

Based on explicit and implicit information, we argue that Székelys possessed detailed knowledge of the local ecological system. Moreover the world’s first known explicit mention of ecosystem services (“Benefits that are provided by Nature for free”) originated from this region from 1786.

## Background

A large number of in-depth studies show that traditional/indigenous/local ecological knowledge can effectively help conserve biocultural diversity and heritage (e.g. [1–3]). Many authors, together with the Intergovernmental Panel for Biodiversity and Ecosystem Services [4], call for greater efforts and new ways to use traditional/indigenous/local ecological knowledge in order to safeguard biodiversity and ecosystem services at different levels.

It is also well established, that besides an in-depth knowledge of the local environment, self-governance by local communities is a powerful way of maintaining a sustainable, resilient social-ecological system (e.g. [1, 5–8]. Ostrom [7] lists, among others (such as collective-choice rules, leadership, norms and social capital), knowledge of the local social-ecological system as vital for a sustainable self-governed system. Researchers argue that resource users should share common knowledge of the local ecological system, and have an in-depth understanding of the local carrying capacity of the resources/ecosystem services [1, 7].

Together with the study of the way that recent self-governed systems functioned, historical studies also enrich our understanding of the value of local ecological knowledge in resource management. An important institution for the management of local resources and ecosystem services in medieval and modern Europe was the village law or village by-laws (e.g. *England*[9], *Denmark*[10]*, Austria*[11], *Germany*[12], *Holland*[13]). Village laws regulated forest and grassland management, especially pasturing and haymaking, the order of cultivation on arable fields, the use of common fields, the use of water resources, communal self-government, the rights of craftsmen, clothing and punishments for stealing and other improper behaviour [5, 9, 10, 13–15]. In Transylvania, as a result of the privileged status of the Székely community, village laws were framed by the whole Székely village community (free Székely peasants, nobles and serfs). As Székely village laws were written by locals to manage the local landscapes, they allow us to reconstruct the contemporary local ecological knowledge, in particular how people understood landscapes, ecological patterns and processes, and how they managed their ecosystem services.

In our paper, we use the term landscape ethnoecological knowledge (LEEK), which is a subset of traditional ecological knowledge (TEK, [2]). As defined by Johnson and Hunn [3], landscape ethnoecological knowledge focuses on the ecological features of a landscape (e.g. ecotopes, habitats and other landscape elements), and shows how the living landscape is perceived, named, imagined, classified and managed by the people who inhabit it. Of the numerous definitions of ecosystem services, the following was used in our paper: ecosystem services are the benefits people obtain from ecosystems [16]. In subsistent-oriented economies, communities depend directly on local ecosystems for food, timber, water and other products needed for their livelihood [2, 17]. We agree with Kumar [18] that we should realize (especially in historical investigations) that the properties of ecological systems that people regard as “useful” may change over time, even if the ecological system itself remains relatively constant. Thus, study of ecosystem services demands parallel analysis of the environment and the socio-economic system.

Village laws have usually been published and analysed from historical, legal and agricultural viewpoints (e.g. [9–12, 14]) but there is a scarcity of analyses from an ecological point of view [13, 15, 19]. Dirkx statistically analysed regulations by focusing on forest grazing by different livestock and other uses of woodlands and used the *Marke Boeken* to reconstruct the deforestation history of the Dutch landscape [13]. Vera, Buissink and Weidema [15] used village laws to document medieval woodland structure and regeneration, forest grazing and masting. Imreh [19] argues that village laws resulted in many cases in the protection of the natural environment. Besides these publications, there are a large number of studies on the late medieval and modern environmental history of Europe. Studies focused on, among others, modelling agro-environmental systems [20–22] and analysis of long-term landcover/landuse-changes and their driving forces [23–26]. Historical overviews of pre-industrial resource management systems are also frequent [27–34]. These and some other results suggest that villagers in medieval and modern time Europe had a profound understanding of the ecology of the surrounding landscape [9, 10, 14, 32, 35]. However, to date, no explicit analysis of their landscape ethnoecological knowledge has been undertaken.

The main aim of our study was to document how Székely village laws regulated the management of the landscape and its ecosystem services during the 16-19^th^ centuries. Both region and the chosen time period are also rich in quantifiable, comparable and relevant historical data. Our specific goal was to reconstruct the landscape ethnoecological knowledge of Székely people based on published village laws [5, 14]. Our hypothesis was that an in-depth ecological understanding of species, habitats, ecological processes and carrying capacity of resources was needed in order to sustain a management system that lasted for at least 300 years.

### Study area

**Figure 1:**
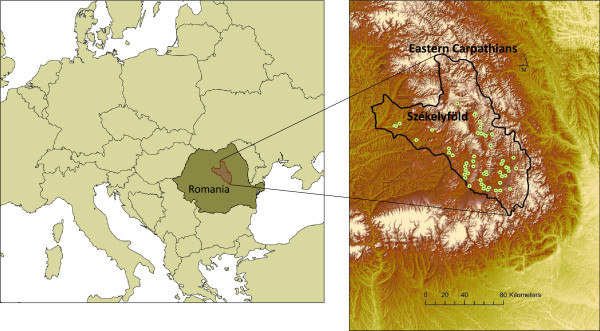
**Location of the study area in Székelyföld, Transylvania, Romania.** Dots show the 52 Székely villages for which village laws were available (map source: ASTER-GDEM, 2009, NASA).

The study area is located in the Székelyföld region of Transylvania, Romania (12 800 km^2^, coordinates: 45°32’- 47°09’ N; 24°24’- 26°26’ E, Figure 1). From the legal historical viewpoint, Székelyföld is the sum of those areas where Székely (pronounced as Se: kei) law was determinative. Like some other mountainous regions of Europe (cf. [36]), the region formed a relatively stable social-ecological system from late medieval times until the Second World War [37]. Small-scale, traditional agriculture was retained in many places, even during socialism (1945–1989).

**Figure 2:**
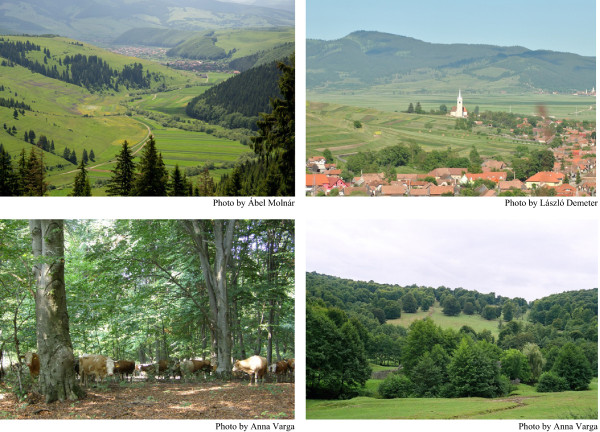
**Typical landscapes of the Székelyföld region, Romania, dominated by spruce forests and meadows, arable fields and villages, beech forests, and pastures.** Although these landscapes are examples of present-day landscapes, they are thought to have many similarities with the landscapes of the 17-18^th^ centuries.

The western part of the study area belongs to the Erdélyi-Mezőség (a highland region, 300–500 metres above sea level, covered by Pannonian and Sarmata deposits), the eastern part belongs to the Eastern Carpathians (a mountainous area, 700–2300 metres above sea level with crystalline bedrocks, flysch and neogene volcanic surfaces). The two main rivers of the region are the Maros (Mureş) and Olt (Olt). Climate is moderately continental with short summers and long winters. The annual mean temperature ranges from 4 to 7°C, the annual precipitation is 500–700 mm in the highland areas and basins and 1000–1200 mm in the mountains. Forests still cover ca. 35–40% of the area. By the 20^th^ century, most oak forests, many beech and some spruce forests had been replaced by arable fields, pastures and meadows. The highland areas are dominated by *Fagus sylvatica*, *Quercus petraea* and *Carpinus betulus* forests, whereas mountainous areas are covered by *Picea abies* and *Fagus sylvatica. Tilia cordata, Fraxinus excelsior* and *Acer* spp. are also widespread (Figure 2). Above ca. 1500–1700 metres, subalpine grasslands and shrubs are typical. Many grassland types and patches in Transylvania are species-rich. Some of the most species-rich dry grasslands in the world [38] and mountainous hay meadows in Europe (80–85 vascular species per 16 m^2^; [39]) are to be found in the region. The main crops of the region are potato and maize. After the building of the railway network in the late 19^th^ century, spruce timber became an important export good. The 52 villages examined, and for which information on village laws was available, are scattered throughout the Székelyföld region.

### The Székely people

Székelys form a Hungarian ethnic group that has lived in this area for at least a millenium. The time period examined in this study (1581–1847) covers the last two and a half centuries of feudalism, namely, the pre-capitalistic period. During this period, most Székelys lived in self-governed village communities. Villages were composed of three main social classes: the upper class with the highest social position comprised the highest nobles (called primor), having moderately-sized estates and owning land in several different villages. They constituted ca. 3 % of Székely society. The second group comprised the lower nobles: constituting ca. 50-60% of the population. These had “noble peasant” status (not paying tax, having rights to elect local village leaders etc.) and served the king as soldiers, providing also services (e.g. food and accommodation) for the army during periods of both war and peace. They are also called the free Székelys. The third major social class was formed of outlawed, farming serfs who had to pay taxes.

At the turn of the 15-16^th^ centuries, human population of the Székelyföld region was ca. 70 000 [40], i.e. ca. 5 people/km^2^, which increased to 15/km^2^ by 1786 and to 24/km^2^ by 1850/1851 [41]. In 1651, on average, 51 families lived in a Székely village [42]. In contrast to Western Europe, the latifundium mainly consisted of scattered lands in Székelyföld. In most cases, the three main social classes shared different proportions of the Székely village-territory. A significant part of the territory was owned by free Székelys. The structure of the society was relatively stable during the 270 years examined [42]. In 1614, 53% of the population was free Székely, 25% was serf, while in 1844/1847, 52% of the population was free Székely and 22% was serf [37].

In the Székely self-government system, the village itself elected its own leader, who was himself controlled by the village community. The task of the elected leader was the organization of the village and its commons (e.g. forest use, grazing, arable farming). The leader was required to make the village community undertake public work and to keep order and discipline. In a Székely village, there was usually constant competition for public benefits, legitimacy, material welfare and positions of power in society. The village community could possess common estate and obtain income, punish collectively, take action or organize village meetings.

In Székelyföld, personal freedom was generally considered of little importance. However collective freedom (freedom of the whole village community) was relatively high. The state had little direct power over individuals compared with neighbouring countries. Village communities were able to buffer against the local impacts of regional/national driving forces [5].

### Székely agriculture and forest management

During the 17-18^th^ centuries, the agricultural techniques of the Székelys were similar to those found in many other European countries. Data show that the 18^th^ century agricultural revolution of Western Europe had not reached the region until the mid 19^th^ century [5]. Since there were just a few large seigniorial domains, small-scale farming played a significant role in agriculture (Table 1). The nobleman’s domain neither became a model nor played a significant role in the changing of traditional practices [5]. Since Székelys were averse to change, development of cultivation techniques was very slow. This is also evidenced by the long (often unchanged for over 100 years) survival of individual village laws [5].

**Table 1:** **Some important data about Székely farming between 1650 and 1750 (Kászonszék region,** [42] **)**

Data calculated per household	Note
2.5 -3 hectares arable fields	
1.2-2.1 horse and ox	35% of the population lack these
0.8-1.8 cow	ca. 1.5 cart of hay per livestock unit
8-10 sheep	
4.8-6.4 cart of hay	=6-10 days using a hand-held scythe
0-2 male children	

Units represent households.

The household was the working unit, while the village community was the institution that oversaw the entire cultivation system.

In the 16^th^ century, hereditary estates began to spread, but even at the end of the 18^th^ century, community land ownership of arable fields, hay meadows, pastures and forests was common in Székelyföld [5]. Owners with higher social position and major personal estate could possess a larger proportion of common arable field, meadow and acorn yield, but had no greater timber rights than others in the prohibited forests [5]. Lack of timber and firewood occurred in many villages, but the distribution of shortage was unequal. According to data from 1808 for a subregion of Székelyföld (Udvarhelyszék), community members were allowed to clear forests in 32 villages, clearing was prohibited in 70 villages (where forests/wood were in sufficient supply), and there was a shortage of satisfactory trees in 26 villages [5].

During the 19^th^ century, forests and pastures were not separated from each other. The border of adjacent villages was also often not strictly defined, especially in the more mountainous areas. Until the 19^th^ century, Székelys owned common regions (called “havas”, meaning alps, mostly subalpine forest-grassland mosaics) on which every settlement could have a claim. Production for the markets started mainly in these common forests at the end of the 18^th^ century [5].

Until the late 18^th^ century, the two-field system was dominant in Székelyföld. In this system, arable and fallow fields were rotated annually. Fields of a village were arranged into an arable and a fallow block. Fallows were grazed. Owing to the extensive hinterland, this form of agriculture was more favourable for animal husbandry (mainly cattle, sheep, horse and pig) [5]. Fodder production on arable land was almost absent.

In 1870, the proportions of land-use types in Kászon (a subregion of Székelyföld) was as follows: 41% forest, 31% hay meadow, 18% arable land, 8% pasture, 2.5% non-used land [42]. Forest cover of Székelyföld, as estimated by Szabó on the basis of the First Military Survey, was approx. 47–48 % at the end of the 18^th^ century [43].

Near the villages, sessile oak forests with hornbeam and beech forests dominated. Pastures, fallow land, forests and hay meadows were used for grazing. Fences prevented free movement and grazing by livestock. Székelys left stubble about a span tall so that weeds could regenerate rapidly, resulting in high quality pastures. As a result of grazing, fallow lands became manured and after 2–3 ploughings, weed density had decreased.

Wheat, barley and oats were the main arable crops. Maize and potato appeared only at the end of the 18^th^ century [5]. There were fruit trees in the gardens and forests, and sometimes grape vines were cultivated on warm hillsides. The intensity of manuring was much less than it was in Western Europe. On average, the Székely household owned ca. 5 livestock units. These animals were kept in the stable or barn from December until February, and produced only ca. 12–13 carts of manure. This amount of manure was not sufficient even for 0.5 hectare of arable land [5]. Rather than cart out manure from the stable, manure production by the corralling of sheep overnight predominated. In the second half of the 18^th^century, the frequency of manuring a single parcel of land was generally once every 6–8 years. During the intervening period, the land was left fallow 3–4 times. Stable manure was only rarely applied to hay meadows [44].

## Methods

### Village laws

The first written village laws were produced in Zalán and Gyergyóújfalu (Zǎlan and Suseni) in 1581. The last village law was written in 1847. At the time of framing these laws, Székelys thought that they had already been in force for much longer [5]. This means that oral forms (perhaps even written forms) of these laws were already common before this time. Some of the centuries-old Székely laws survived until present and are still used in pasture and forest commons, as in other European countries (e.g. Italy, Portugal, Sweden, Scotland, France, Spain, the Netherlands) where commons have also survived until present [33, 45–47].

Village laws laid down the will and intention of local community members from generation to generation. Village laws were written down by local or appointed clerks, but the regulations were framed and approved by the whole community. Székelys believed that an adequate village law must be both old enough and sufficiently projected into the future [5]. There was no unified Székely village law; all of them were unique, but because of similarities in terms of landscapes, societies and economics, they have much in common. Village laws were announced from time to time, mainly for the sake of new community members [5]. In Western Europe, community rule-making and jurisdiction had already been suppressed by the 16^th^ century. However Székelys started to commit their ancient laws to writing at that time. Székelys often emphasized in their laws that the judge could only make decisions “with the support of the community”.

A typical Székely village law consisted of 2–5 pages comprising 5–20 short or long regulations. In the first introductory part, the Székely people stated the intention of the law. Here, they usually referred to respect for ancestors and inherited responsibility. This part was followed by the regulations which referred not only to the use of forests, grasslands, arable fields etc. studied in this paper, but to other spheres of village life, such as the punishment of criminals, obligation of the judge, the order of fire-fighting and postal service etc. In the last part, some community members would testify about the accuracy of the law and the consensus of the villagers.

It is important to emphasize that the laws of each village were based entirely on local, inside knowledge of the communities: they were based on former written and oral laws, customs and common laws and the perception of changing conditions. There is no evidence that the laws were copies or that they were adapted from those of other villages. Terms used in village laws testify to their independence [5].

During the Enlightenment, the Habsburg Monarchy deliberately reduced village self-government. However, by the second half of the 18^th^ century, the Monarchy had still not been able to dominate local village life. Although state forest law already existed (1781), it was only partly effective in this region. By the 19^th^ century, the system of village community had become obsolete and a hindrance to the process of modernization. Nevertheless, open-field systems could be seen in many places until the commencement of World War II.

### Data collection

When collecting data, we used printed publications as sources of village laws. These included: “The self-regulating Transylvanian village” [5] and “Order in the Transylvanian village” [14]. We examined every village law published in these volumes (52 Székely villages, 72 village laws). Laws were written during the 16-19^th^ century in a form of the Hungarian language that is still comprehensible today. We grouped and interpreted regulations according to the categories of the DPSIR framework, sorted them into a table, and then encoded and aggregated the data.

We used the DPSIR framework to understand, analyse and quantify the Székely social-ecological system in its complexity. The DPSIR framework was developed by the European Environmental Agency (EEA) in 1999 in order to be used as a general platform for environmental data collection, categorization and dissemination [48]. According to Bürgi et al. [49], the DPSIR framework is particularly useful for the evaluation of planning processes.

We paid particular attention to any ecological information that emerged either explicitly or implicitly from village laws and to the tacit traditional ecological knowledge. We collected the local folk names of every animal and plant species, the categories of folk habitats, habitat mosaics, types of forest and grassland uses and we analyzed the ecological context of the regulations. The latter was supported by our botanical and ethnoecological studies that have been conducted in this region since 2000 [39, 50].

Since it was the contemporary landscape ethnoecological knowledge that formed the focus of our attention, we did not reconstruct the actual monetary value of the punishments that were meted out (e.g. Florints, Florenis, Flor., Denars). The value of the currency might have also changed throughout the 17-18^th^ centuries [5].

### Data analysis

The DPSIR concept has been widely used for documenting and understanding environmental problems and developing preservation strategies [51, 52], and rarely for analyzing historical landscape management systems [53]. Driving forces in the DPSIR framework are forces that elicit and define those human activities that relate to the use of landscapes and ecosystem services. The main driving forces can be socio-economic, political, technological, natural and cultural [49], and can be global, regional or local in scale. In our study, we paid particular attention to the local social demands (see also [26, 29, 54]). Driving forces result in different pressures. Our study focuses on pressures relating to human activities, such as clearing and management of forests, use of pastures and hay meadows, arable lands and water, in general the use of ecosystem services. The state of the natural environment may change in response to pressures. Species composition, dominant species, tree age and density, nutritional value of the grass cover and soil fertility all reflect the quality of ecosystems. Impact is usually perceived as a reduction or a shortage of ecosystem services (e.g. less timber) caused by changes to the environment. This encourages the community to respond. These responses are intended to solve reductions and shortages caused by pressure-induced changes.

For the appropriate use of the DPSIR framework, it was essential to understand the meaning behind certain passages thoroughly. Hence for the analysis, we used the footnotes and writings of Imreh and other contemporary source publications (eg. statistics, litigious cases, village community decrees, decisions and resolutions; [5, 14, 37, 40–42, 44]).

We quantified not only driving forces relating to pressures, but also driving forces relating to responses. While the former is defined as the demand of the active executor of a certain pressure action (e.g. cutting of a tree for personal use), the latter is defined as the demand of the community (e.g. preserving large enough tracts of forests for future use by the community). This is not about driving forces on a personal and community scale (cf. [54]), rather the judgement of a certain action can differ for each of the two social scales. As the statements were often partly implicit owing to the concise framing of village laws, we reconstructed both implicit and explicit driving forces.

We divided the responses into two main groups: (1) responses limiting the use and supporting the regeneration of ecosystem services; and (2) responses that protect produced/available ecosystem services from theft and destruction, thus ensuring their fair distribution among community members.

As well as presenting a quantitative analysis of our database, we show the style and ecological content of village laws, together with original quotations.

Finally, we reconstructed all types of landscape ethnoecological knowledge that appeared either explicitly or implicitly in Székely village laws according to DPSIR categories. Knowledge related to obtaining and managing ecosystem services was considered to be Pressure-related knowledge. Knowledge related to the perceived usefulness of ecosystems was considered to be Impact-related knowledge, that is, knowledge resulting from State-related knowledge and monitoring, which helps in recognizing actual or potential changes in the condition of resources. Indeed, for appropriate interpretation of impacts, it was necessary for villagers to recognize what the exploitable ecosystem services actually were and which ecosystem services or functions had diminished or were about to diminish.

Response-related ecological knowledge was considered to be the knowledge related to the maintenance, regeneration, and the prevention of deterioration of ecosystem functions and services used by the community. Finally, we considered as Driving force-related the knowledge that informed decisions on what expectations and demands for ecosystem services could be met by the landscape.

## Results

**Figure 3:**
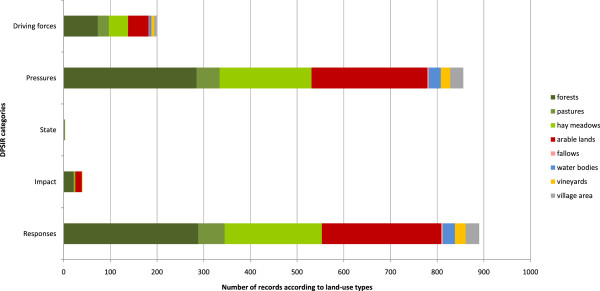
**Frequency of records of DPSIR categories mentioned in Székely village laws arranged according to land-use types.**

A total of 898 ecologically relevant individual regulations were found. Owing to their nature, village laws referred mainly to pressures and responses (856 and 890 records, respectively, e.g. in connection with the felling of trees, mowing and grazing, Figure 3). Székelys rarely accounted explicitly for the necessity of their laws in terms of state and impact (only 2 and 40 records, respectively). The large number of explicit references to driving forces was surprising (199 records).The greatest number of records (sum of all DPSIR categories) related to forests (674), followed by arable land (562), hay meadows (448) and pastures (134). Village area (62), water bodies (57) orchards/vineyards (50) and fallows (7) were referred to only rarely (Figure 3).

Székelys used and managed several ecosystem services, many of which were explicitly referred to in village laws (Table 2).

**Table 2:** **Ecosystem services managed by Székely village communities in the 16-19** ^**th**^ **centuries based on explicit information in village laws**

Ecosystem services*	Records in laws
Cultivated crops (cereals, vegetables, domestic fruit)	192
Wild plants and their produce (wild fruit for food)	16
Wild animals and their produce (freshwater fish, crayfish, birds, game)	15
Fibres and other materials from plants for direct use or processing (wood, timber and all other sorts of timber uses)	110
Materials from plants for agricultural use (grass for forage and fodder, acorn)	217
Surface water for non-drinking purposes (domestic use, washing, cleaning, soaking hemp)	19
Plant-based energy resources (wood fuel)	22
Animal-based energy (physical labour provided by horses and oxen)	39
**Total**	**630**

*Categories follow CICES classification of Ecosystem Services vers. 4.3 [55].

### Driving forces

Village laws contained a total of 22 different types of driving forces. The most important driving forces were local: requirements for timber and firewood, food, forage and hay (Table 3).

**Table 3:** **List and sum of driving forces (explicit + implicit) in pre-capitalistic Székely village laws**

Type of driving forces	Pressure-related driving forces	Response-related driving forces	Translated texts of quotations from Székely village laws
			(explicit pressure-related driving forces)
1. demand for wood (not specified)	136	277	“4 trees per capita are given for the need of community members” “When one fails to join the group extinguishing fires, he shall pay 20 Denars.” [5: 234]
2. demand for timber	24	0	“When a community member has the intentions to build, he must report his will to the court and ask for permission to cut trees down; a slip is given to him containing the amount, the species and the location of the trees to fell.” [5: 454]
3. demand for firewood	16	1	“When herdsmen herd cattle to the forest, they are allowed to collect lying dead trees (except oak) for fire in prohibited forests.” [5: 307]
4. demand for lumber and wood for tools	12	0	“When one cuts apple or pear trees that is not his own tree for sheep or for any other reason (even for tools), he shall be fined 3 Hungarian Forints.” [5: 387]
5. demand for wood for carting	6	1	“Wood for spoke, wheel and hub can be cut down to meet the needs of community members.” [5: 328]
6. demand for oak or beech bark	5	0	“It is forbidden across the whole region (both on meadows and in forests) to fell beech trees for stripping or timber and to ring-bark them.” [5: 497]
7. demand for wood for broom making	4	0	“They not only pruned the trees but they also cut them down. When one prunes or cuts birch trees down in prohibited forests from this time on, he shall be fined 3 Hungarian Forints.” [5: 473]
8. demand for wood for charcoal burning	2	0	“Blacksmiths can only use stumps in prohibited forest for charcoal burning. When one resists, he shall be fined 3 Hungarian Forints.” [5: 473]
9. demand for wood for lime-burning	1	0	“It is forbidden to burn lime in the community forests. With the permission of the community, it is however legal to burn lime under certain circumstances.” [5: 398]
10. other demands for wood (specified)	15	1	“Birch, poplar and hornbeam trees can be cut down under certain circumstances for minor needs, but only in the agreed, limited quantity.” [5: 315]
11. demand for forage	517	72	“When one grazes his cattle deliberately in standing hay, he shall be fined and furthermore, must pay for the damage.” [5: 440]
12. demand for hay	98	220	“When hay meadows are closed for grazing (after 24^th^ April), it is prohibited to mow on the meadows. Otherwise the fine is 12 Florenis.” [5: 358]
13. food demand	102	298	“When one collects fruit or vegetables from the gardens of other people without permission of the owner, he shall be fined 40 Denars” [5: 349]
14. demand for baking and brandy making	4	10	“It is forbidden to place the distiller cauldron in an endangered area.” [5: 395]
15. demand for water rich in fish and crayfish	0	6	“No one shall dare to fish with harpoon or *Verbascum* in the water of the River Olt.” [5: 319]
16. demand for clean water	3	18	“As we need river water for our living… it is forbidden to throw garbage, manure or any carcass into or next to the river or into the streets.” „Some of the community members open gates or leave gaps in the fences for their cattle to go to the river, causing damage to fellow members.” [5: 374]
17. demand for clothes and leather	8	0	“When painters pollute the common living water of the community or butchers wash the intestine of cattle in the river, they shall be fined 40 Denars.” [5: 347]
18. demand for linen	4	2	“It is permitted to keep retting-ground lakes (for hemp) in certain places, but it is forbidden to keep lakes to the disadvantage of the ditches of mills or water bodies, otherwise he shall pay 3 Florines fine.” [5: 374]
19. necessity of waste dumping	13	0	“When one throws garbage or manure into the streets, he shall be fined by the community 50 Denars.” [5: 367]
20. demand for clean village	0	3	“Throwing garbage or weed into the street is fined 1 Florine.” [5: 438]
21. demand for transportation	3	3	“When one intends to make a path, he must report his intention at the community meeting.” [5: 300]
22. financial needs	52	0	“It is strictly forbidden to transport or sell timber or firewood to other villages.” [14: 113]

Distinction was made between driving forces relating to pressures and responses. Original quotations show how Székely people perceived and used different ecosystem services. Numbers in brackets indicate data source and page numbers.

**Figure 4:**
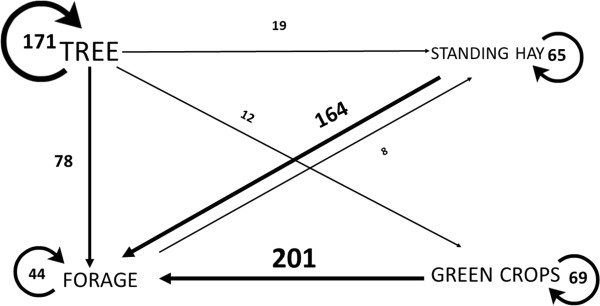
**Competition for ecosystem services (only the main ecosystem services are shown).** Arrows mark the activities related to the use of ecosystem services. Endpoints of arrows mark the explicit or implicit purpose (driving force relating to pressure, mostly the interest of the user), starting points mark the damaged or illegally used ecosystem service protected by the village law (i.e. driving force of the response, mostly the interest of the owner or the community). Where there are circular arrows, the two driving forces are the same. Numbers represent the total records found in village laws.

The relationship of pressure- and response-related driving forces, i.e. the competition for ecosystem services is shown in Figure 4. Data show that conflicts mainly relating to the grazing of green crops and standing hay, as well as overuse of forests, must have been regulated in 16-19^th^ century Székely villages (Figure 4).

Some 56% of the 930 pressure-related driving forces (both implicit and explicit) were related to the feeding of animals (Table 3, entries 11, 12) and 11% was due to the food demands of humans (Table 3, entry 13). Another 24% indicated the need for wood for self-sufficiency (Table 3, entry 1). Financial needs (market and trade) formed a relatively small proportion (only 5.6%) of driving forces (Table 3, entry 22). Almost 40% of the need for wood was explicitly specified in the laws (e.g. timber, firewood, tools, stripping or broom making, Table 3, entries 1–10). In total, we found 55 different uses for wood.

### Pressures

#### Forests

The most frequently regulated pressure relating to forests was the felling of trees (83%) (Table 4, entry 1). Székely people used not only timber, but also firewood, wood for tools, lumbers and carts. The overuse of oak is explicitly referred to several times (Table 4, entry 2). Grazing activities formed 14% of pressures relating to forests. Cutting of leaf-fodder was referred to relatively rarely (Table 4, entry 10), but we know from other sources that it formed a significant pressure, and hence was forbidden [5]. Székelys protected forests not only from the grazing of leaves, branches and buds by livestock, but also even from herdsmen “herding with axes in their hands”. Another pressure was the burning of forest litter (Table 4, entry 9) which was in the interest of herdsmen (dead grass disappeared and fresh grass grew). Fire damaged trees, and so burning was forbidden by the community. As oak acorns and beech masts were usually produced only once every 10 years (sometimes, however, in large quantities [5, 44]), many villages regulated masting (Table 4, entry 7) (Székelys masted mainly beech, see also [44]). Since the regional distribution of beech and oak was uneven, one village would often mast the territory of another. Some villages prohibited masting by foreign pigs.

**Table 4:** **Pressures related to forests, pastures, hay meadows and arable fields in Székely village laws**

Regulated pressure	Total	Prohibition	Regulation	Fine	Other penalty	Quotations from Székely village laws
**Forests**						
1. felling of trees (without mention of species)	166	121	45	88	89	“When one fells living or dead trees in prohibited forests, he shall be fined 3 Florins.” [5: 341]
2. felling of oak tree without permission	23	16	7	12	12	“Felling of oak trees is most harmful and dangerous, thus forestguards must also take care of them in the village.” [5: 454]
3. felling of beech tree without permission	10	7	3	4	5	“When one strips or fells a fruiting beech or oak tree, he shall be fined 3 Hungarian Forints.” [5: 306]
4. bark stripping	7	5	2	4	3	“It is forbidden to strip oak trees in prohibited forests and likewise in open-to-use common forests.” [14: 113]
5. collecting of dead wood	3	2	1	1	2	“It is permitted to collect lying dead trees and branches of dead trees, but it is obligatory to report this intent to the owner of the forest.” [5: 346]
6. ring-barking of trees	15	11	3	6	10	“It is forbidden to ring-bark trees in the escarpment forests.” [5: 497]
7. masting foreign or too many pigs	11	11	0	8	5	“When God gives acorn/mast, it is obligatory for the community to set up a guard. It is forbidden to mast foreign pigs without permission, otherwise flor 3.” [5: 372]
8. forest burning	5	5	0	3	2	“It is forbidden to burn the common forests. If somebody resists, they will be fined 6 Hungarian forints.” [5: 300]
9. burning of forest litter	5	5	0	3	3	“When one burns forest litter, he shall be fined according to the law, for 1 Forint in unbound forest.” [5: 437]
10. leaf-fodder cutting	4	4	0	4	0	“Herdsmen with sheep and goats grazing in the forest during winter are forbidden to carry an axe or to cut branches. If a herdsman resists, he shall be fined 3 Forints.” [5: 497]
**Total**	**249**	**187**	**61**	**133**	**131**	
**Pastures, hay meadows and arable fields**						
11. driving livestock into green crops or standing hay	200	153	47	148	85	“It is forbidden to drive cattle to fields, hay meadows belonging to other people until the liberation of the fields.” [5: 462]
12. leaving gate open/damaging gate	112	50	62	95	31	“When one leaves a gap in his backyard fence or damages the fence, he shall be punished.” [5: 354]
13. fastening horse/cattle to pickets	7	1	6	3	5	“When one works in the fields, he must fasten his cattle to pickets. If damages were caused by them, he must be fined 25 Denars and furthermore, pay the damage.” [5: 376]
14. herder or community member housing foreign cattle/sheep	24	22	2	15	9	“It is strictly forbidden for anybody of any kind of rank to receive foreign cattle and graze them around the village.” [5: 382]
15. grazing hay meadows after Saint George’s Day	8	8	0	5	3	“It is forbidden to keep cattle in the hay meadows after Saint George’s Day.” [5: 279]
16. grazing during night	6	6	0	3	3	“It is forbidden to keep cattle in the fields after sunset. When one resists, he shall be punished.” [5: 298]
17. grazing sheep/goat/goose in pastures for beasts of burden	6	6	0	2	4	“It is forbidden to make a sheepfold in a cattle pasture. When one resists, he shall be fined 1 Forint.” [5: 361]
18. grazing livestock (other than ox and horse) inside the village fence	2	2	0	2	0	“From Saint George’s Day until Michaelmas, it is forbidden to graze any animal other than beasts of burden and milking cows returning home within the village fence. The fine is 40 Denars.” [5: 363]
19. conversion of pastures into hay meadows	5	5	0	1	4	“It is forbidden to transform common pastures into hay meadows.” [14: 175]
**Total**	**472**	**348**	**124**	**323**	**210**	
**Other land-use types**						
20. washing dirt into water	11	11	0	8	3	“When one washes manure or other dirt into the stream, he shall be fined 1 Forint.” [5: 367]
21. catching crayfish/fishing in prohibited streams	7	6	1	4	3	“When one fishes in the communal stream, in Babora stream, Lebed stream or Köd stream, if caught, he will be fined by the community 3 flor.” [5: 327]
22. draining water of hemp lakes into the stream	3	3	0	2	1	“It is obligatory to take care of hemp lakes. If the water of a hemp lake drains into the stream, the fine is 100 Denars.” [5: 408]
23. diverting stream into gardens	1	1	0	0	1	“In order to keep the stream clean, it is forbidden to divert it into the gardens.” [Imr5: 361]
24. digging the bed of river/stream	4	0	1	3	1	“It is obligatory to clean the stream bed every year, thus every village member has to take part in the digging, otherwise 100 Denars.” [5: 408]
25. felling of wild fruit trees (even if it is as thin as a stick)	6	1	0	5	3	“If someone fells fruit trees, whether pear or apple trees, even in their own forest, even if the tree is as thin as a stick, he shall be fined 50 Denars per tree.” [5: 325]
26. collecting fruit	6	5	1	4	3	“Trees and fruits yielded in the forest or meadow are under strict prohibition. Thus, it is forbidden to sell or even donate them to outsiders, otherwise 1 Hungarian Forint.” [5: 412]
27. collecting unripe fruit/crop	1	1	0	1	1	“Some people collect unripe wild apples and often damage the branches. It is forbidden to collect wild apples before Michaelmas, otherwise the fine is 1 Forint and the cart and cattle of the delinquent must be confiscated.” [5: 329]
**Total**	**39**	**28**	**3**	**27**	**16**	

Prohibitions and regulations were counted separately, as much as monetary and other kinds of penalties. Original quotations show how Székely people perceived and communicated pressures.

#### Pastures and hay meadows

Village laws distinguished between types of grazing based on species, age and the sex of animals, as well as the circumstances and the location of grazing (Table 4). Different regulations can be observed in connection with grasslands differing in type and quality. Usually, cattle took priority over sheep. In some places, stubble fields, in others, fallows, and in most cases, forest cattle pastures were protected from sheep (Table 4, entry 17). Hay meadows among the fallows were used as cattle pastures and protected against digging by pigs pastured there. Separate pastures were maintained by the communities for beasts of burden (ox, horse) and protected against other livestock (Table 4, entry 17). In the afternoons and at night, beasts of burden were herded into the forests to graze fresh grass [5]. The grazing of standing hay (Table 4, entry 11) belonging to others was another commonly prohibited activity. This is the reason why it was also prohibited for individuals to remain outside the village at night (Table 4, entry 16). Punishments differed according to whether the damage was accidental or intentional.

Since the winters were long, early spring pastures were a scarce resource. Therefore, livestock was allowed to graze the early growth of hay meadows until Saint George’s Day (24^th^ April). In autumn, owing to early frosts in subalpine regions, the unmown second growth was also grazed after Michaelmas (29^th^ September). Székelys only rarely cut the second growth on hay meadows and manuring was very rare, even at the beginning of the 19^th^ century (cf. [44]). Subalpine shepherds were often hired from other villages. Village laws ordered them to stay as long as possible in the subalpine region with the sheep. It was forbidden for herders and community members to receive foreign cattle or sheep without the consent of the community (Table 4, entry 14). Surprisingly, there was no reference to herding- and guard-dogs (protection against wolves and bears) in village laws.

#### Arable fields and fallow lands

The grazing of green crops was the pressure most commonly indicated as a source of damage (Table 4, entries 11–13). The amount one was fined reflected the damage caused to green crops: between 29^th^ September and Christmas, the fine was 40, until Carnival it was 10, until Easter it was 20 and until harvesting it was 60 units of the local currency [5]. However, grazing on spring cereals, mainly when there was a shortage of hay during poor springs, was permitted. Sown fodder and grass did not exist during the 17-18^th^ centuries in Székely villages. We hardly found any data referring to bush and forest encroachment on grasslands and fallow lands. Sometimes, shrubs were eradicated from fallow lands that had been abandoned for 10–50 years [5].

Grazing by beasts of burden whilst working on the fields also caused problems in that it could provide opportunities for stealing. Since it was necessary to feed the beasts of burden at the scene of the agricultural work, there were strict regulations for this (cattle and horses had to be tied to pickets, Table 4, entry 13).

Fences were placed around the village and between green crops and grazed fallows. It was obligatory to keep the gates shut. Many regulations related to gates (112 records, 24% of records in Table 4, entry 12). Sometimes, Székelys strengthened the fences by planting willows.

#### Other pressures

As water was regarded a common resource, it was also protected by regulations. Streams were protected against pollution and overfishing (Table 4, entries 20–23). Fishing was prohibited altogether in some places, while fishing rights were accorded only to nobles in other regions. In some villages, fishing was unrestricted, but data for this are scarce. Regulations relating to stream beds (Table 4, entry 24) are referred to surprisingly rarely, and there are no data for floods.

Even less data refer to hunting e.g. of wolves and bears. The shooting of wolves and bears was rewarded. More data are available for the beginning of the 19^th^ century. At that time, hunting was permitted in most Székely villages (e.g. deer, hare, fox, birds) [44].

As it formed the common property of the communities [5], the collecting of fruit from fruit trees (mainly apple and pear) was strictly regulated in many villages (Table 4, entries 26 and 27), and the felling of wild fruit trees was strictly prohibited (Table 4, entry 25). The collecting of other non-timber forest products (listed in [44] for the early 19^th^ century) e.g. wild fruit (*Vaccinium* spp., *Fragaria* spp., *Rubus idaeus, R. fruticosus* agg., *Corylus avellana*) and oak-gall, the honey of wild bees, medicinal plants and fungi was not referred to in village laws.

### States and impacts

States and impacts of ecosystem functions and ecosystem services, as well as changes to these, were rarely mentioned in village laws (only 2 state and 40 impact records, respectively).

Explicitly mentioned impacts related mainly to the excessive decline in forests and pastures: “forests are so overused that shortly, it will not even be possible to find firewood in our forests”; “Despite our old laws, the beech-covered peaks inherited from our forefathers have almost been destroyed…unless we forsee these problems (and act accordingly), we shall neither find timber nor firewood in these forests”; “If ring-barking of forests continues, we will have to obtain the required wood from other villages.”; “the area for cattle pasture is too small”; “due to the overuse of forests…because of fallen trees, cattle and sheep could not graze freely”.

Despite the scarcity of explicit state records, we found many different local folk names for habitats, animals and plants in village laws (Tables 5 and 6). We found 71 different folk names for habitats (including vegetation types, vegetation-related land-cover and land-use categories, 676 records). Habitat categories named after a (dominant) plant species were only found in the case of forests and arable lands (e.g. beech forest, wheat stubble) (Table 5).

**Table 5:** **Folk habitats, habitat mosaics, land-use and land-cover types mentioned in Székely village laws**

Folk habitats, habitat mosaics, land-use and land-cover types in Hungarian	Records	English equivalents
határ	122	territory of the village (incl. houses, fields, forests etc.)
mező	31	cultivated area
vetésmező	3	arable area (crops)
nyomás, nyomásmező	7	fallow field
erdő	87	forest
szálas tölgyerdő	1	high oak forest
csereerdő, csere	6	oak forest
makkos erdő	3	oak forest (with acorns)
bükkös erdő, bükk	2	beech forest
fűzberek, füzes, csigolya	3	willow grove, willow shrub
szabad erdő	7	open-to-use common forest
tilalmas erdő	24	protected forest
öreg erdő, eleven erdő, nyers erdő	4	old, little-used forest
legelő	4	pasture
puszta	2	open area
pázsit, pásint	5	grass (embedded in arable land)
havas	16	mountain area (pasture and forest)
legeltetőhely	1	grazing field
ökörlegeltető hely	1	ox pasture
marhalegelő	1	cattle pasture
esztenahely	1	sheep pasture
kaszáló, kaszálóhely	21	hay meadow
szénafűhely, szénafű, fű	79	hay field
rét, szénarét	23	meadow
erdőn lévő kaszáló	3	meadow in/near a forest
irotván szénafű	1	cleared for meadow
havasi kaszáló	2	alpine meadow
sarjú, sarjútarló, torló	18	place with second growth (meadow and stubble)
pallag, parlag, mezei parlag	11	old field
gyep	6	grasslands among arable field
patak	12	small stream
patak árka	3	ditch of a stream
patak martja	1	bank of a stream
tó	1	pond
kenderáztató tó	1	hemp pond
folyóvíz, víz	7	river, stream
Olt mejéke	1	floodplain of the Olt river
nád	1	reed bed
vetés, őszvetés, tavaszvetés	29	green crop, autumn crop, spring crop
szántóföld, föld	15	arable field
gabona, gabonás	47	cereal field
búza	6	wheat field
tarló, gabonatolló, búzatorló	4	stubble, wheat stubble
zabhatár	1	oat field
törökbúzavetés	1	corn field
veteményes	2	vegetable field
borsó, mák, pityóka, káposzta, répa, hagyma	10	pea, poppy, potato, cabbage, mangel beet, onion fields
ugar	2	fallow field
csóvás parlag	1	signed old field
föld vége	3	field margin
mesgye	2	field boundary
út, út mellett	11	road, road verge
szőlő	8	vineyard
szőlő lábja	1	lower margin of a vineyard
gyepű, véggyepű, oldalgyepű, gyepű széle	3	different grassy, bushy field boundaries
faluközt	2	among the houses in the village
telek	1	plot
kaszálókert	1	fenced meadow
csűrkert	1	barnyard
kert mellett	1	along a fence
jószág	2	a property

**Table 6:** **Wild plant and animal species mentioned in Székely village laws (original quotations)**

Local and Latin names	Records	Quotations from Székely village laws
*csere* (oak, *Quercus petraea* and *Q. robur*)	29	“It is prohibited to graze goats during winter in the diminished oak forest of the mentioned village. Otherwise the fine is 6 Forints.” [5: 401]
*bükk* (beech, *Fagus sylvatica*)	13	“It is prohibited to ring-bark beech trees. When one fells it, he must carry it away. But it is prohibited to ring-bark and leave the tree on the spot.” [5: 300]
*nyár* (poplar, mostly *Populus tremula*)	7	“Birch, poplar and hornbeam trees can be felled under certain conditions for small needs, but only in the established limited amount.” [5: 315]
*gyertyán* (hornbeam, *Carpinus betulus*)	2	“All trees we have in Gelye Árnyéka beech, birch, poplar and hornbeam, except oak and alder in the stream, can be felled for the need of community…when one trades trees felled from that place or gives them to foreign villages, price of the trees shall be turned to the benefit of the village.” [14: 114]
*nyír* (birch, *Betula pendula*)	10	“Gypsy broom- and spoon-makers caused serious damage to birch and poplar trees and not only pruned the trees but also felled them for brooms. When someone prunes or fells the birch trees of prohibited peaks, his punishment shall be 3 Hungarian Forints.” [14:125]
*kőris* (ash, *Fraxinus excelsior*)	1	“Wood for spokes, wheel and hub and ash trees, can be felled to meet the needs of community members.” [Imreh 1983: 328]
*éger* (alder, *Alnus glutinosa* and *A. incana*)	3	“The felling of birch and alder trees in these prohibited peaks carries a fine of 3 Hungarian Forints.” [5: 472]
*fűz, csigolya* (willow, *Salix fragilis* and bushy *Salix* spp.)	4	“When one cuts a willow belonging to other people, he shall be fined 50 Denars.” [Imreh 1983: 342] “Every community member is obliged to plant at least 12 willow trees among fences in order to protect the territory.” [5: 358]
*maszlag (mullein, Verbascum* spp.)	1	“Nobody should even try to fish with harpoon or *Verbascum* in the water of River Olt.” [5: 319]
*körtvélyfa, körtvény, körtövély* (pear tree, *Pyrus communis* and *P. pyraster*)	3	“The felling and damaging of fruit-bearing pear, apple and cherry trees grown in free forests and meadows is subject to similar prohibition.” [5: 372]
*almafa* (apple tree, *Malus domestica* and *M. slyvestris*)	3	
*cseresznyefa* (cherry-tree, *Cerasus avium*)	1	
*hal* (fish, *Pisces*)	9	“When fishermen, gypsies or vagrants fish here, their catch shall be taken away and they shall be expelled.” [5: 429]
*rák* (crayfish, *Crustacea*), mostly freshwater lobster *(Astacus sp.)*	2	“We decided and prohibited the catching of fish and crayfish in Dimén stream, Uzon loka and Pisztrongos, otherwise the fine is 1 Hungarian Florine” [5: 387]
*medve* (brown bear, *Ursus arctos*)	1	“When one shoots wolf or brown bear either in the forests or meadows of the community, he shall recieve 3 Rft for wolf shooting and 6 Rft for bear shooting from the common money of the community.” [5: 477]
*farkas* (grey wolf, *Canis lupus*)	1	

In total, we found only 4 names for wild animals and 12 for wild plant species (13 and 77 records, respectively). There was no mention of *Acer* and *Tilia* species or shrubs in village laws (Table 6).

While domectic animals were often mentioned (sheep 45 records, goat 13, ox 24, cattle 167, milk cow/calf 9, horse 20, pig 15, goose 3), cultivated plants were mentioned less frequently (wheat 14 records, maize 11, oats 4, poppy 8, pea 11, onion 1, cabbage 2, potato 2, hemp 4, lentil 2, bean 4, squash 1, fodder beet 1).

### Responses

Most of the responses related to forests (Table 7). Székelys had many ways of protecting their forests (Table 7). Often, certain wood states (Table 7, entry 1) and species (Table 7, entry 2) were gathered or felled in certain amounts (Table 7, entry 4) on certain dates (Table 7, entry 3) and for certain purposes (Table 7, entry 12), but only after permission had been granted (Table 7, entry 6). Marketing (Table 7, entry 8) and use by outsiders (Table 7, entry 9) were often strictly regulated. Valuable forests were occasionally managed: nursing oak trees, thinning poorly grown beeches and shrubs, thinning out dense forests, removing useless wood from forests.

**Table 7:** **Responses limiting the use and supporting the regeneration of ecosystem services and responses protecting ecosystem services and their fair distribution (with examples)**

Responses	Forest	Pasture	Meadow	Arable	Fallow	Water	Fruit, grape	Other	Total
**Responses limiting use and supporting regeneration**									
1. permitted only in certain state (of wood, animal)	12	5	-	2	-	-	-	-	19
2. permitted only in the case of certain species (of wood, animal)	28	7	2	2	-	-	-	-	39
3. permitted only from/until given date (grazing of hay meadows, collecting of wild apple)	-	6	18	16	1	-	1	2	44
4. permitted only in certain amount (felling of trees, grazing of cattle)	14	1	1	-	-	-	1	1	18
5. permitted only on certain days (felling of trees, harvesting)	5	-	-	1	-	-	-	-	6
6. permitted only with permission/report (felling of trees, receiving of foreign sheep, selling of hay)	38	9	15	19	-	-	-	1	82
7. price according to the value (wood, grazing cattle)	9	-	4	3	-	-	-	-	16
8. cannot be marketed from the village (wood, hay, corn, fish, fruit, grape)	23	-	3	1	-	1	4	-	32
9. outsider not permitted to use local ecosystem services (acorn, pasture, hay)	11	15	3	-	2	-	2	-	33
10. must be protected against damage (putting out of forest fire, pollution of water)	2	1	-	-	-	13	-	-	16
11. permitted only in certain places (felling of trees, mowing, grazing of sheep/cattle)	103	16	59	42	-	4	-	4	228
12. permitted only for certain purposes (for tools, for sick livestock, for the need of the community)	6	-	1	1	-	1	1	-	10
13. absolutely prohibited (felling of trees, grazing, fishing)	31	3	-	3	-	3	-	3	43
14. rewarding (revealing of delinquent, shooting of wolves and bears)	2	-	-	-	-	-	-	1	3
15. permitted only by certain means ( grazing by fastening to pickets)	1	2	1	-	-	-	-	1	5
**Total**	**285**	**65**	**107**	**90**	**3**	**22**	**9**	**13**	**596**
**Responses protecting ecosystem services and their fair distribution**									
16. community permitted to, individuals not permitted to (fell trees, graze, mow)	3	1	1	-	-	-	-	1	6
17. prohibited to steal from individuals (wood, hay, crop, fruit)	2	-	8	13	-	-	3	11	37
18. prohibited to steal from the common property (wood, hay, crop, fruit)	2	-	5	5	-	-	-	-	12
19. prohibited to damage property of other members/community (forest, pasture, hay meadows, arable lands)	71	5	85	122	-	1	9	14	307
20. damage prevention (gates must be closed, grazing in the forest only without carrying an axe, cleaning of river/stream bed)	27	2	49	64	-	6	4	3	155
21. permitted only at a certain time of the day (felling of trees, mowing, harvesting)	2	-	5	6	-	-	-	-	13
22. protected by a hayward (forest, flock, hay, green crops, grape)	5	2	15	18	-	-	-	1	41
23. permitted	1	-	1	-	-	-	-	-	2
24. must be shared fairly (wood, grass)	3	-	1	-	-	-	-	-	4
25. other	3	-	-	1	-	1	-	-	5
**Total**	**119**	**10**	**170**	**229**	**0**	**8**	**16**	**30**	**582**

In the case of pastures, regulations focused on seasonal rhythms, the quality of forage and the ranking of different animals (by species, age, state). Furthermore, Székely people also took the nature of the property (private or common) and the remoteness of the place into consideration. Grazed forests were protected against goats and the cutting of leaf-fodder. The least number of responses was found in the case of fallows (Table 7); uncropped parts were grazed relatively freely. In the case of hay meadows, Székely people attempted to prevent the grazing, trampling down and stealing of hay.

In regulations relating to green crops, the prevention of direct stealing and damage caused by trampling and grazing played a major role. Here, different punishments were meted out according to the species, age and sex of the grazing animal, as well as to the circumstances (accidental or deliberate) and place of grazing [5].

Haywards, who protected crops, hay and trees, played an important role in the enforcement of rules (Table 7, entry 22). Over the years, every member of the community had to honour this commitment, with 4–20 guards protecting these resources, both by night and day [5]. If someone resisted doing his duty, he would be fined and forced to fulfil his responsibility. If haywards could not present the offender who had caused the damage, they had to pay for the latter. Haywards were paid both a wage and a reward (one- or two-thirds of the fine!) [5].

The most common form of punishment used for the regulation of pressures was a penalty (Table 8), but compensation and distraint were also common. Physical punishment, arrest, decimation, payment in kind and being banned from the use of the ecosystem service occurred rarely. Intentional and repeated damage or damage caused by outsiders were punished more strictly. The penalty was often considerable; it could be as much as half the price of an ox.

**Table 8:** **Forms of punishments mentioned as responses in Székely village laws**

Forms of punishment	Forest	Pasture	Meadow	Arable	Fallow	Water	Fruit, grape	Other	Total
penalty	167	24	140	174	1	18	13	28	565
payment in kind	1	1	2	1	-	-	1	-	6
compensation	11	1	30	35	-	-	2	2	81
physical punishment	2	-	-	-	-	-	-	-	2
distraint	8	-	8	12	-	-	2	-	30
banning out of the use of ecosystem service	2	1	3	2	-	1	-	-	9
arrest	1	-	-	-	-	-	-	-	1
decimation	-	2	-	-	-	-	-	-	2
punishment according to the law	18	2	6	13	-	-	-	1	40
not mentioned	100	30	51	57	2	7	10	9	266
other	1	-	-	-	-	-	-	-	1
**Total**	**311**	**61**	**240**	**294**	**3**	**26**	**28**	**40**	**1003**

## Discussion

### The management of the local ecological system

The main (often explicit) purpose of the framing of the Székely village laws was to promote the reproduction and regeneration of ecosystem services and to prevent shortage in these services. A strong sense of “self-awareness” can be seen in village laws. Székelys drew up regulations for themselves, introduced prohibitions and ordered behaviour and type of activity based on their ancient values in order to keep in check the selfishness of individuals [5]. Székelys argued that a long past and local framing legitimized the laws. The variables often encountered in functioning commons (e.g. collective-choice rules, leadership, norms/social capital and knowledge of the social-ecological system, [7]) were also present in our system, though we did not analyse these in detail.

Written regulations were regularly adjusted as environment and society changed (cf. adaptive management [56]) and were often improved as new situations arose. Sometimes the Székelys framed new laws: “Human thoughts are changing, thus laws must change; it is necessary to adapt them to fit the spirit of the times” [5].

The ecological knowledge and wisdom of previous generations were often incorporated and cited by explicitly referring to the positive or negative experiences of ancestors. In several cases, Székely people openly mentioned also the needs of the following generations in their village laws (“The forest called Akasztófa will never again be sold but will be grown on for the next generation.”). Imreh argues that the aim of village laws was “to create states that provide safety through constancy for the next generations” [5]. Székely villagers were motivated and forced to become more moderate, careful and self-controlled [19].

The remarkably fine-tuned regulations were diverse in form. The common forms of responses were prohibitions, restrictions and activities that could be done only with permission. In our particular case, the three main fields of regulation of commons (cf. [8–10]) were also noticeable, namely: restrictions on grazing, limiting the felling of trees and regulating water supplies and sources. Székely village communities were controlled systems based on punishments similar to their Western European counterparts [9, 10, 13, 15]. The fine and conscious regulation of private and common properties in village laws is illustrated by the planter of fruit trees: these not only had the right to the produce of the tree, but retained this right even after the field had changed hands [5].

Responses restricting the use and supporting the regeneration of ecosystem services and responses protecting produced/available ecosystem services and ensuring their fair distribution among community members also illustrate awareness of regulations and a deep understanding of ecological processes. The fine-tuned adaptation of regulations to changing social and environmental conditions necessitated constant monitoring of the state of the environment. In most cases, Székelys were not short of some ecosystem services, whereas in others, the shortage did not cause any problems (e.g. fungi, certain wild fruits, wild game, fish in several cases). These were not regulated by village laws. It is interesting that spruce and the regulation of its use were never mentioned in village laws.

In our opinion, the reason for the low number of state records in village laws was that it was necessary only to express those states in village laws that had strong negative effects on the community. Although it is possible to identify and quantify impacts without any positive or negative connotations, merely simply by recording a change, we discovered that only strong negative impacts had been recorded. Thus, the list of explicitly-stated impacts gave a distorted picture, since only really dramatic situations requiring state reinforcement were recorded in village laws. However, implicit reference to impacts was common: e.g. there was/there would be insufficient timber, firewood, forage, fodder, fish for the future.

During the 270 years examined, the strength of regulations relating to forests, hay meadows and arable land-use increased [5]. This tightening of regulations usually related to overexploitation caused by trade and to the intensification of agriculture, which was becoming increasingly privately owned. Village laws show a remarkably interwoven and carefully organized system of cultivation. The village community made decisions about which plant should be cultivated and where, the time at which a community member was compelled to undertake certain agricultural activities and when and which part of the village territory was to be used for or protected from grazing.

Many regulations showed that there was serious competition for ecosystem services. Personal and communal interests conflicted. For example, the use of slowly regenerating wood conflicted with the short-term use of forage and leaf-fodder, whereas, in other cases, green crops and hay were protected from grazing for personal goals. Data showed that village laws could effectively restrict the destructive impact of grazing on communal forest land. Clearing of forests that resulted in an increase in the area of arable land and hay meadows was supported by the community, but clearing for personal purposes was prohibited or, at least, strictly limited. Communities limited seigniorial clearings above all. According to many village laws, trees could not be sold to outsiders. However, commoditization of resources spread during the late 18^th^ century and, as a consequence, the areas of village forests in close proximity to big towns and markets had diminished considerably by the 19^th^ century [5].

Village regulations gave a long list of forest uses. The large number of uses recorded (55 types) is comparable to the 50–90 types of wood products found by Johann [29] for Austria and the 61 types of forest uses documented by Bürgi et al. [57] for Switzerland.

During the 19^th^ century, the Székely village community endeavoured to resist the expansion of the Monarchy administrative system. The interests of the Habsburg Monarchy and that of the Székely communities were different: while the Monarchy would seek to transform forests close to the village into taxable arable land, communities sought to sustain them as forests [5]. The Székelys argued that they could protect ecosystem services using locally framed laws and regulate their use [5].

Székelys aimed for fine-tuned forest use by promoting regeneration and by maximizing the felling of trees. The awareness of laws is shown by the many explicit messages that refer to long-term changes in forest quality. Their goal was to provide wood for the “rest” (next generations) in the long run, and to promote continuous renewal of the forests, while keeping them suitable for grazing (free forests were grazed relatively freely). The main goal was to suppress private grazing which could cause further damage. The grazing of livestock in herds was easier to organize and control [5]. Regeneration of valuable forests was promoted not only by instituting prohibitions over their use, but sometimes even by active forest management. Modern methods of forest regeneration, e.g. renewal of forests from seed or the introduction of alien tree species were, however, never mentioned. We found that the most frequently mentioned problematic pressure was the felling of oak. According to village laws, oak was the most valued tree, as it was in many other regions of Europe [9, 13, 15, 33, 35]. Székelys, by means of their laws, sought to substitute the felling of oak with that of less valued species (spruce, beech, dead trees and certain pioneer species). Also, the presence of hornbeam may have required special attention during the regeneration of oak forests, as hornbeam could outcompete oak when regeneration was poor (cf. [58]). Felling of spruce forests was never regulated explicitly. These forests were mainly far from villages, mostly in the subalpine regions, and were still common during the 17-18^th^ centuries.

Management of pastures and meadows was less regulated. The main goal of village laws was to reduce overuse near villages and prevent theft (working in the fields was a good pretext to feed the animals and on some occasions, people deliberately drove their cattle, with silenced bell, into cereal fields, while drawing aside and pretending not to do so intentionally [5]). Spring-time cleaning of hay meadows (e.g. collection of leaf litter and twigs, removal of ant hills – a common practice nowadays in the region, [39]) was not mentioned in village laws. Although sheep yielded significant profit [5], the more valuable grasslands were protected from them. In adapting to local conditions, sheep were pastured in subalpine regions, where grass was poorer.

In the 18^th^ century, one grain of wheat, on average, yielded three grains [5]. Thus, it is conceivable that under these circumstances both theft and prevention of theft were equally important. Regulation of theft and other forms of improper behaviour demonstrated that at a personal level, Székelys were less moderate, whereas village law regulations indicated that at the community level, long-term thinking was dominant.

In summary, we conclude that Székelys framed responses by adapting well to the local, slowly changing internal and external driving forces, i.e. they applied adaptive management [56]. Imreh argues, however, that by the 19^th^ century, the open two-field system framework had become too strong [5]. The strictly applied common organization of agricultural activities gave little room for a higher degree of adaptive management based on individual needs (e.g. more optimal manuring of arable lands for more efficient maintenance of soil fertility).

Increased marketing and trading at the end of the 18^th^ century onwards, as well as financial needs, resulted in communities dividing common land: “land was no longer a working birthright and the source of food for families, and that which maintained communities. Lands became properties that caused their owners to drift with business-like thoughts and money-making aims towards market” [5].

### Spatial and temporal scales of village laws

Székelys regulated the use of ecosystem services on five spatial scales: individual trees (fruit trees and trees in prohibited forests), the plant community (mostly in the case of forests), the habitat mosaic (especially in arable-grassland and grassland-forest mosaics), the village territory (inner and outer parts of the territory) and sometimes even on a broader scale (common territories of several villages).

The village territory provided the space in which the life of the Székely village took place, and thus, where its regulations applied (landscape ecological scale). The ‘ecosystem’ (sensu [36, 59]) was therefore the basic unit of management.

Regulations showed that the balance of land-cover categories was fine-tuned. For example, the clearing of forests near the village and the conversion of pastures into hay meadows was not permitted. We argue that Székelys regulated consciously the proportion of arable land, pasture, meadow and forest area at this landscape ecological scale in order to maximize the long-term exploitation of ecosystem services provided by the local landscape. There are data available on similar optimized land-use structures from other regions of the Carpathian Basin dating from the Middle Ages [32]. We found that management on an even coarser spatial scale was also present in some cases, e.g. in common forests and common alps of several villages, and transhumance by Romanian herdsmen.

Areas near the village reached and sometimes exceeded their full capacity, but there were also reserves (sometimes significant) in the outer territory. Knowledge relating to the limits of exploitation was essential in regulating land-use type and intensity. The main spatial scale was the habitat mosaic. Areas dominated by arable land, oak forests and grasslands with high productivity near the village differed from that dominated by mountain spruce forests and subalpine outer pastures. A fundamental principle of the regulations was to protect the inner territory against overuse, and to relocate certain uses to the outer territory (cf. [33]). For example, herdsmen had to “go far away”; subalpine pastures were often leased out to herdsmen from neighbouring villages; inner forests and pastures were more subject to prohibition; the ring-barking of trees was often permitted in the outer forests, but not close to the village. Székelys also fine-tuned the grazing of subalpine regions and inner pastures: livestock was sent to the subalpine regions as soon as they could be grazed there and could only return when there was insufficient grass on the mountains. Thus, biomass exploitation of the inner and outer sub-systems was connected. Furthermore, Székely people protected hay meadows embedded in fallows, grasslands embedded in arable lands and hay meadows located between grazed forests. The importance of the habitat mosaic scale was shown also by the 71 different types of habitat and habitat mosaic names mentioned in village laws.

A finer spatial scale (the level of the plant community) was observed explicitly only for forests. We assume that the management of pastures and hay meadows was less adapted to the patchiness in vegetation, but data are limited. The well-developed and fine-scale management of hay meadows in the 20^th^ century was documented for the nearby Gyimes region, e.g. scattering of hayseed, rotation of parcels of land, management of wet or mossy patches, selective eradication of certain species, eradication of *Nardus* from certain areas by corralling sheep for the night [39], but there are no data relating to the time period in which these fine-scale practices developed in the Székelyföld region.

The area of pastures was not defined in village laws and management of pastures was regulated only at a landscape scale. We assume that herdsmen possessed plant community level ecological knowledge (daily and seasonal grazing routes were probably adapted to the spatial pattern of more or less useful species and to the grazing preference of livestock, (cf. [60])). However this was not made explicit, nor was it implicit in the regulations. It was generally true that village regulations rarely referred to specific uses of wild plant species (except to those of trees).

The finest scale regulated was that of individual plants. Cutting was prohibited or strongly restricted in case of fruit trees and trees of prohibited forests.

The temporal scale of village laws, even explicitly, was very large. On the one hand, they had existed “from ancient times”. On the other, they were meant “for descendants”, “for the coming generations” and even “for eternity” [5]. Thinking on the century time scale occurred only at this general level and concrete, ecological phenomena at this level were not mentioned. Changes during the latter decades, e.g. reduction in ecosystem services were referred to, often concretely, mainly for forests, and rarely for pastures. Regulation of management at the decade time scale could only be observed for forests (prohibited parts of the forests until they had matured). At the end of the 18^th^ century, owing to the effect of the Enlightenment and capitalism, short-term planning started to spread in community management, and the effect of trade and individual goals became stronger [5].

### Landscape ethnoecological knowledge of Székelys

Based on our analysis, we reconstructed the presumed landscape ethnoecological knowledge of the framers of village laws (Table 9). Based on explicitly expressed or implicitly inferred knowledge, we argue that Székelys possessed detailed knowledge of the local ecological system, the past, the dynamics and the managing possibilities of the landscape and available ecosystem services.

**Table 9:** **Reconstructed landscape ethnoecological knowledge of Székely villagers related to driving forces, pressures, states, impacts and responses based on the analysis of 16-19** ^**th**^ **century village laws**

Topics of traditional ecological knowledge	Mentioned in village laws	Not mentioned/missing
*Driving force-related knowledge*		
fine-tuning of proportions and types of land uses according to the needs of the community and adjusted to the productivity of ecosystem services	optimization for husbandry, relatively little arable lands, equilibrium of arable lands and hay meadows and pastures and forests, proportion of cattle and sheep, proportion of subalpine and inner pastures, need for oak and old trees, necessary number of beasts of burden, forests for reserve, liberation of territory at an optimal date (stubble, second growth)	-
fine-tuning of ecosystem service use to the regeneration potential scaled to one household for free/money	number of trunks/carts of wood, amount of arable lands by ’arrow draw’, number of pigs that can be masted, sometimes no fish for peasants	pasture area needed per livestock unit, need of livestock unit per household
sensible use and improvement of landscape potential (e.g. soils, climate, relief)	mountains as obstacles, living “as our ancestors lived”, “sowing of fodder is the invention of room scientists”	weather
*Pressure-related knowledge*		
finding ecosystem services in the landscape	knowledge of the distribution of forests and pastures with different qualities and usefulness, locality of wild fruits	distribution of non-woody wild plant species, wild fruits, medicinal plants and fungi
maintaining, managing and increasing ecosystem services and related ecosystem functions, knowledge of the effect of human management factors on the decrease and increase of services	hardly mentioned, usually without explanation e.g. nursing forests, clearing of forests, grazing	ring-barking, manuring of arable lands and meadows, cleaning of hay meadows, weeding, pasture maintenance
“harvesting” ecosystem services	felling of trees, mowing, grazing	harvesting crops, collecting fungi
*State-related knowledge*		
knowledge of species and habitats	trees and cultivated species, habitats	wild herbaceous and shrub species
knowledge of vegetation dynamic processes, succession and regeneration processes, changes of ecological conditions	profound knowledge of forest regeneration	regeneration of grasslands, changes in weed composition and density
knowledge of the landscape, orientation in the landscape, knowledge of different “localities”	local knowledge often occured explicitly (toponymes), also knowledge of the neighbouring village territories	regional knowledge of the far landscape
knowledge of past states of the landscape, monitoring of landscape changes	often mentioned, but mainly generally and in the case of forests, mainly based on a decade time scale	changing state of grasslands, knowledge of century scale landscape history
*Impact-related knowledge*		
monitoring of actual states of ecosystem functions and services (e.g. trees, edible species, cultivated plants, productive soils)	timber, firewood, wood for tools, pastures, hay meadows, wild fruit trees, cleanness of waterbodies	fungi, other than woody wild fruits, medicinal plants, famine foods
recognition of demands for exploitable ecosystem services, recognition and prediction of potential changes in services	see the list above	see the list above
*Response-related knowledge*		
fine-tuning of exploitation of ecosystem services to the regeneration rate of ecosystem functions (prohibitions, limited/regulated or free uses)	increased protection of slow-growing tree species and fruit trees, prohibition of cutting of leaf-fodder, prohibition of ring-barking, protection of young trees, sparing of inner pastures, protection of streams from pollution	overgrazing of grasslands, regeneration of grasslands, fungi, etc.
tuning of the degree of punishment to the value and the regeneration potential of the damaged ecosystem service	fine is greater in the case of the felling of oak than for other tree species, fine is greater for grazing green crops than for the grazing of standing hay, unbound forests are free	overgrazing, fungi, etc.
the effect of regulation on the ecological state, and thus on the maximum possible exploitation rate of the local ecosystem services	grazing rank of livestock (ox, cattle, sheep, pig), felling of living/dead trees, grazing of hay meadows before Saint George’s Day and after Michaelmas	use of pastures, fungi, etc.

Village laws were most abundant in Driving force-related and Response-related LEEK. In the case of pressure, state and impact, it was often difficult to reconstruct the mostly implicit local ecological knowledge. The small number of state records would imply that every member of the community had knowledge of the good/bad ecological conditions of the ecosystems of the village territory. While ecologically harmful activities were often emphasized in village laws (the prohibition and restriction of some activites), ecologically favourable activities were rarely mentioned. We argue that the latter might have been common knowledge. It would seem that almost every member of the community had a basic level of LEEK, and regulations were founded on it. This LEEK was not possessed by outsiders and new immigrants to the same degree. A profound knowledge of the landscape, the nuanced naming of places with toponyms, perception of changes and a knowledge of the degradation and regeneration processes of ecological functions and services could often be identified in the handing out of legal sentences. Responses drafted to prevent decline and degradation also demonstrated an in-depth knowledge of ecological processes. In general, a more detailed ecological knowledge was observed for forests than for grasslands.

Unfortunately, some aspects of LEEK were so well known within Székely communities that they were not made explicit in village laws (see the last column of Table 9). Other significant parts of LEEK remained implicit because they were not related to restrictions. For example, the Székelys’ in-depth understanding of fungi was not apparent in village laws but is shown by their more recent nuanced and still traditional knowledge of different species and their habitat preferences in the region (15–35 folk fungi taxa /village) [61].

It was particularly surprising to find more folk habitat names than names for wild animal and plant species in village laws. Székely regulations mostly focused on sites, habitats and not on species. The reason for this may be that in village laws the use of certain sites and habitat types was regulated, and Székelys knew from the toponyms that the name referred e.g. to a place dominated by oaks.

Comparison of the habitat types mentioned in Székely village laws with both recent and historical folk habitat names documented in Gyergyó and Gyimes regions [50, 62] revealed a great overlap (e.g. of main forest types and land-use types), but a conspicuous lack of reference to spruce forests, spring fens, reed beds and stony-rocky places. Presumably, there was no need to regulate the use of these habitats.

Although Székely people presumably knew the dominant grasses and herbs of pastures and hay meadows, surprisingly none of these nor the weed species of arable fields were mentioned in village laws. According to data collected by Rab [62] and Babai and Molnár [50], 150–250 wild plant species are known by locals in the villages of this region. As most of these names occur in medieval sources and toponymes of medieval or earlier origin [62] we argue that the absence of wild plant (and animal) names from village laws does not imply a lack of knowledge of these species by Székelys.

### The DPSIR framework

The DPSIR framework used in our analysis helped towards the identification, structuring, quantification and assessment of the local ecological knowledge related to key socio-economic drivers, pressures, states, impacts and local responses, thus providing a holistic and comprehensive approach to the complex issues relating to the sustainable management of natural resources. The DPSIR framework helped especially to locate implicit ecological knowledge, including part of the ecological knowledge embedded in planning and practice (see Table 9). Previously the DPSIR framework was most often used to describe rapidly deteriorating environmental situations. We demonstrated that the framework is also suitable for the analysis of long-term human-nature relationships occurring within a relatively stable socio-economic system.

## Conclusions

The Székely village law is a good example of the sustainable way of thinking and of the conscious maintenance of ecosystem services. Our analysis showed that laws were based on ecological principles and were adapted to the local landscape. Village laws were written for the benefit and survival of the community, for the protection of public benefits, and for the maintenance of ecosystem services [5, 19].

Moreover, we suggest that the world’s first explicit mention of ecosystem services was worded in this region (cited in [19]). In 1786, a local Székely official in Sepsiszentgyörgy (Sfântu Gheorghe) wrote the following about firewood, timber and wood for tools (see the original Hungarian text in Table 10). “The usefulness, both specific and general, of the Regulation of Forest, was intended to provide necessary benefits for the Human Community, and this is evidenced by the everyday uses of wood; namely for the daily fire, for necessary protection and shelter, and for the tools which are necessary for general living. Some members of the community abuse these *Benefits that are provided by Nature for free* (emphasis added by the authors), by the inappropriate, premature felling of acorn-bearing trees and trees suitable for timber, and this causes obvious harm to our descendants” (cited in [19]). Similarly, in another Latin language document dating from 1787 the expression *„inaestimabili naturae beneficio”* was used [63]. These data predate the findings of Mooney and Ehrlich by nearly 80 years (published in 1864 by G.P. Marsh, cited in [64] see also [65]). The early data demonstrates the conscious management of the natural environment by the Székelys.

**Table 10:** **Probably the world’s first documented explicit reference to ecosystem services (cited in** [19] **)**

Original text in Hungarian	Translation of the original text into English
“Micsoda szükséges jókat akarván az Emberi Társaságban, és micsoda hasznos légyen – mind különösen mind pedig közönségesen – az Erdőnek Conservatioja, azt megbizonyítják a mindennapi fával való élések; nevezetesen a mindennapi **tűz**, minket elfedező **hajlékunk** szükséges volta és járás-kelésre nézve elkerülhetetlenül megkévántató szükséges **eszközök**. Ezen, **Természet ingyen való Jovaival** pedig mely igen visszaéljenek a lakosok – a makktermő és épületnek való fáknak helytelenül, ideje előtt való leerdőlésekkel – a következő posteritásnak igen nagy praejudiciumára, nyilván vagyon.” [19]	“The usefulness, both specific and general, of the Regulation of Forest, was intended to provide necessary benefits for the Human Community, and this is evidenced by the everyday uses of wood; namely for the daily **fire**, for necessary protection and **shelter,** and for the **tools** which are necessary for transportation. Some members of the community abuse these **Benefits that are provided by Nature for free,** by the inappropriate, premature felling of acorn-bearing trees and trees suitable for timber, and this causes obvious harm to our descendants.” [19]

We conclude that Székely village laws served the long-term interest of the local community over shorter-term personal interests and also against the Habsburg Monarchy, and helped the conscious and sustainable management and protection of ecosystem services for at least 300 years.

We documented that the Székely community had great adaptive capacity to deal with changes in the ecosystem services provided by the local landscape, as well as with the increasing pressure of external driving forces. This locally regulated resource management seems to be crucial in the long-term conservation of landscape and biological diversity, and it has likely been a key factor in the unique state of preservation of Transylvanian biodiversity (cf. [29, 38, 66]).

The cultural and biodiversity-preserving role of the long-term application of stable, subsistent-orientated systems is widely acknowledged [2, 39, 66]. Research and, if possible, resilient maintenance of these systems is extremely important, especially in a time when biodiversity loss and global change are having a profound impact on Europe, as well as other continents [46, 66–68]. We agree with Scotti and Cadoni [47] and Fischer et al. [66] that direct links between local communities and local landscapes are vital to the maintenance of functioning landscapes, local biodiversity and cultural heritage. Székely village laws are able to provide us with a rich source of ideas for sustainable landscape and resource management.
